# Neurodegenerative diseases and neuroinflammation-induced apoptosis

**DOI:** 10.1515/biol-2022-1051

**Published:** 2025-02-25

**Authors:** Shi Huang, Yaxin Lu, Wanzhen Fang, Yanjiao Huang, Qiang Li, Zhiliang Xu

**Affiliations:** School of Clinical Medicine, Wannan Medical College, 241002, Wuhu, Anhui, China; School of Pharmaceutical Sciences, Wannan Medical College, 241002, Wuhu, Anhui, China; School of Stomatology, Wannan Medical College, 241002, Wuhu, Anhui, China; Human Anatomy Experimental Training Center, School of Basic Medical Science, Wannan Medical College, 241002, Wuhu, Anhui, China; Department of Human Anatomy, School of Basic Medical Science, Wannan Medical College, 241002, Wuhu, Anhui, China; Anhui Province Key Laboratory of Basic Research and Translation of Aging-Related Diseases, Wannan Medical College, Wuhu, 241002, Anhui, China

**Keywords:** neurodegenerative diseases, neuroinflammation, signaling pathways, apoptosis

## Abstract

Neuroinflammation represents a critical pathway in the brain for the clearance of foreign bodies and the maintenance of homeostasis. When the neuroinflammatory process is dysregulate, such as the over-activation of microglia, which results in the excessive accumulation of free oxygen and inflammatory factors in the brain, among other factors, it can lead to an imbalance in homeostasis and the development of various diseases. Recent research has indicated that the development of numerous neurodegenerative diseases is closely associated with neuroinflammation. The pathogenesis of neuroinflammation in the brain is intricate, involving alterations in numerous genes and proteins, as well as the activation and inhibition of signaling pathways. Furthermore, excessive inflammation can result in neuronal cell apoptosis, which can further exacerbate the extent of the disease. This article presents a summary of recent studies on the relationship between neuronal apoptosis caused by excessive neuroinflammation and neurodegenerative diseases. The aim is to identify the link between the two and to provide new ideas and targets for exploring the pathogenesis, as well as the prevention and treatment of neurodegenerative diseases.

## Graphical abstract

**Figure:**
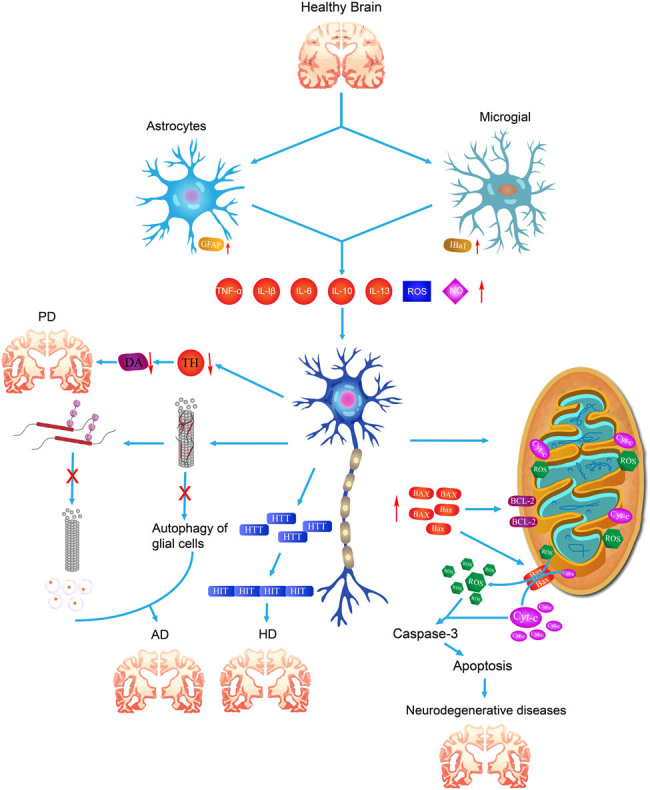

## 1 Introduction

Neurodegenerative diseases are defined as conditions characterized by the gradual loss or deterioration of neurons and myelin sheaths, leading to impaired function over time. A number of neurodegenerative diseases are commonly encountered in clinical practice, including Alzheimer’s disease (AD), Parkinson’s disease (PD), Huntington’s disease (HD) and amyotrophic lateral sclerosis (ALS), among others [1]. Neurodegenerative diseases constitute a substantial threat to human health and quality of life. For example, AD, the most prevalent neurodegenerative disease globally, is estimated to affect approximately 55 million individuals worldwide, with over 10 million cases in China [2,3]. The incidence rate is 5–6% at the age of 65, 10% at the age of 70, and 48% at the age of 90. The age of onset is earlier. It is projected that by 2050, the global prevalence of patients will reach 152 million [4]. As indicated by the World Health Organization, neurodegenerative diseases may become the second leading cause of mortality in humans by 2040 [5]. Neurodegenerative diseases not only inflict significant suffering on patients but also place considerable economic and psychological burdens on families and society. Currently, however, there is no effective treatment for neurodegenerative diseases. The pathogenesis of these diseases is complex and still controversial, and an in-depth understanding of the pathogenesis of neurodegenerative diseases will help to discover new therapeutic targets and drugs, providing new treatment ideas and theoretical foundations for the diagnosis and treatment of neurodegenerative diseases.

It is becoming increasingly clear that neuroinflammation may play an important role in the pathogenesis of neurodegenerative diseases that cannot be ignored. In the brain, the occurrence of inflammatory reactions can result in oxidative stress and damage to the antioxidant defense system of nerve cells, thereby accelerating the progression of neurodegenerative diseases [6]. Concurrently, neurodegenerative diseases can also result in aberrant protein accumulation and the release of inflammatory mediators, thereby establishing a vicious cycle. Additionally, inflammatory cells are capable of secreting neurotoxins such as glutamate, which can result in neuronal overexcitement and subsequent damage. Moreover, inflammatory factors can also stimulate neuronal apoptosis signaling pathways, ultimately leading to neuronal death. Consequently, the inflammatory response is intimately associated with the progression of neurodegenerative diseases, manifesting not only in the initial stages of the disease but also progressively worsening as the disease progresses. Inflammation is a protective mechanism of the body that maintains the internal environment of the brain in a balanced state by repairing, regenerating, and removing damaged histiocytes or infectious agents, toxins from the body [7–10]. Nevertheless, the role of inflammation in the process of organismal aging is also significant. It is a concomitant response to cellular senescence and organismal aging [11–13]. During the aging process, the functionality of the immune system is disrupted due to a deterioration of the body’s immunological defenses. These defenses play a pivotal role in the eradication of pathogens. Consequently, when they weaken, both the innate and acquired immunity systems of the organism become compromised. This results in an imbalance in immune system functioning, which in turn affects the ability of the immune system to clear pathogens, damaged tissues, and senescent cells. Consequently, there is increased expression of pro-inflammatory cytokines (e.g., tumor necrosis factor-α [TNF-α], interleukin [IL]-1β, IL-6, IL-8, reactive oxygen species [ROS]) and C–C chemokine ligand-regulated factors (CCL-2 and CCL-5), which contribute to the inflammatory response [14,15] (Table 1). The chronic stimulation of these factors not only results in a chronic, low-grade, microinflammatory senescent state of the organism, but also induces neuroinflammation and leads to neuronal damage, ultimately resulting in age-related neurodegeneration (Table 1) [16–19].

**Table 1:** Neurologic diseases and neuroinflammatory factors associated with neuroinflammation

Disease	Inflammatory factors	References
AD	TNF-α, IL-1β, IL-6, Tau, NFT	[14,15,22–30,76–82]
PD	TNF-α, IL-1β, IL-6, NLRP3, Iba-1, GFAP, iNOS, COX-2	[14,15,22–28,31–35,116–118,178,179]
HD	TNF-α, IL-1β, IL-6, HTT	[14,15,22–28,36–39,121–136]
Multiple sclerosis (MS)	IL-1β, IL-6, CXC1, CCL2, CCL3, CCL4	[14,15,40–42]
Traumatic brain injury (TBI)	TNF-α, IL-1β, IL-6, IL-8, IL-10, ROS, GFAP	[14,15,43–46]
Gulf war diseases (GWI)	IL-1β, IL-6, IL-2, IL-10, IFN-γ, IL-4, IL-5, IL-17A, IL-33, TSPO	[14,15,47–51]
ALS	G-CSF, IL2, IL15, IL17, MCP-1, MIP1α, TNF-α, VEGF	[14,15,52–55]

Neuroinflammation is defined as an inflammatory response within the central nervous system (CNS) that involves intricate interactions between a multitude of immune cells, factors, and receptors, both in its occurrence and in subsequent development. The principal immune cells involved are microglia, astrocytes, macrophages, T cells, and B cells. The principal factors involved are cytokines, chemokines, ROS, nitric oxide (NO), and prostaglandins. Furthermore, the receptors involved in the inflammatory response include pattern recognition receptors (PRRs) and chemokine receptors (CCR).

Microglia are resident immune cells within the CNS. In physiological conditions, microglia facilitate brain development, repair cellular damage, and promote neuronal survival, thereby maintaining the internal environment of the brain in a state of homeostasis. In pathological conditions, microglia are overactivated by disease factors, which results in excessive inflammatory responses within the brain. This results in the release of inflammatory cytokines and the inhibition of nerve regeneration, which collectively exert neurotoxic effects [20,21]. As individuals age, the misfolded proteins, cellular debris, and other inflammatory stimuli accumulated in the brain lead to the continued stimulation of microglia, thereby accelerating the aging process. Furthermore, elevated levels of organismal senescence result in a reduction in the phagocytic capacity and monitoring abilities of microglia, thereby initiating a self-perpetuating cycle that stimulates the production of inflammatory substances detrimental to neuronal health and facilitates the development of neurodegenerative diseases [22–24]. Consequently, neurodegeneration resulting from neuroinflammation plays a role in the progression of neurodegenerative diseases. The role of microglia-mediated neuroinflammation as a hallmark of several CNS diseases, including AD, PD, and HD, is now well-established (Table 1) [25–28].

A search for inflammatory factors related to several neurodegenerative diseases with high incidence rates or significant adverse effects on human health revealed commonalities in early neuritis. As illustrated in Table 1, an increase in the expression of inflammatory factors, including TNF-α, IL-1β, IL-6, Tau, and NFT was observed in AD [29,30]. A noteworthy increase in inflammatory factors, including TNF-α, IL-1β, IL-6, NLRP3, Iba-1, glial fibrillary acidic protein (GFAP), iNOS, and COX-2, was observed in PD [31–35]. In HD, it has been demonstrated that the expression of inflammatory factors such as TNF-α, IL-1β, IL-6, and HTT exhibited a notable increase [36–39]. In MS, there is a significant increase in the levels of IL-1β, IL-6, CXCL1, CCL2, CCL3, CCL4, and other factors [40–42]. A number of inflammatory factors, including TNF-α, IL-1β, IL-6, IL-8, IL-10, ROS, and GFAP, have been demonstrated to be significantly elevated in individuals with TBI [43–46].GulfWar illness (GWI) has been linked to notable elevations in the levels of various cytokines, including IL-1β, IL-6, IL-2, IL-10, IFN-γ, IL-4, IL-5, IL-17A, IL-33, TSPO, and others, which have been demonstrated to exhibit significant increases in a number of cases [47–51]. A number of factors, including G-CSF, IL-2, IL-15, IL-17, MCP-1, MIP-1α, TNF-α, VEGF, and other factors have been identified in the context of ALS research [52–55].

Neuroinflammation is a critical element in the pathogenesis and progression of neurodegenerative disorders. In recent years, there have been significant advancements in research on the factors, pathways, and cell fate associated with neuroinflammation in neurodegenerative diseases. This article will discuss and summarize the research progress of neuroinflammation in typical neurodegenerative diseases from several perspectives, including neuroinflammation, neuroinflammatory signaling pathways, and changes in cell fate caused by neuroinflammation. The objective is to provide an understanding of the molecular mechanisms of inflammation that contribute to the occurrence and development of neurodegenerative diseases, and to offer insights into potential molecular targets and strategies for the treatment of neurodegenerative diseases at the neuroinflammatory level.

## 2 Neuroinflammation in the most prevalent neurodegenerative diseases

### 2.1 AD

AD, the most prevalent neurodegenerative disease worldwide, which affects over 44 million individuals. Its pathogenesis is complex and plays a pivotal role in the development of dementia. The primary pathological processes of AD include the deposition of amyloid-β (Aβ) protein, hyperphosphorylation of Tau protein, and the production of neurofibrillary tangles (NFTs) [56–60]. The recent discovery of AD-related inflammatory markers, as well as the finding that some innate immune-related genes are also associated with the pathogenesis of AD, suggests that neuroinflammation also plays an important role in the pathogenesis of AD [61–63]. The principal immune cells in the brain are astrocytes and microglia. Neuroinflammation in AD is also mainly related to these cells. Microglia play a pivotal role in the brain, with the capacity to be activated in order to regulate homeostatic balance within the brain when stimulated [64–66]. Some studies have indicated that microglia are abnormally activated in the brains of patients with AD, and this phenomenon may be related to the pathogenesis and development of AD [67]. The development of this state is influenced by a variety of factors, including brain injury, infection, or other stimuli. Upon stimulation, microglia can be divided into two main phenotypes: anti-inflammatory and pro-inflammatory [68–72].

Some studies have indicated that in the context of aging, microglia exhibit a proclivity toward a pro-inflammatory phenotype [73,74]. Upon activation to the anti-inflammatory phenotype, microglia release anti-inflammatory factors, including IL-10, IL-13, and others to counteract the inflammatory response in the brain [75]. When microglia are activated to the pro-inflammatory phenotype, their capacity to remove toxic substances and waste products is diminished, resulting in the accumulation of neurotoxins such as Aβ protein in the brain. Concurrently, the discharge of inflammatory mediators such as TNF-α, IL-1, and IL-6 is enhanced, which in turn facilitates the occurrence of neuroinflammation, accelerates neurodegeneration, and inflicts damage upon neurons within the brain. The diminished clearance capacity of pro-inflammatory microglia results in the accumulation of Aβ protein and NFTs, which are formed by hyperphosphorylated Tau protein, in the brains of AD patients [76–81]. The accumulation of Aβ protein and NFTs may act as a stimulus to continue to activate microglia, thereby creating a vicious cycle and accelerating the development of AD [80,81].

Astrocytes and microglia exhibit analogous functions and are capable of recognizing Aβ, thereby undergoing activation and alterations in morphology and function. Both cells are capable of regulating synapse formation, but the interaction between astrocytes and neurons is bidirectional. Astrocytes and oligodendrocytes are interconnected in the brain, forming a large syncytial glial network comprising hundreds of cells. This occurs through the formation of a tripartite synapse, which involves the connection with neurons. After activation, astrocytes can be classified into two distinct phenotypes, A1 and A2. The A1 type is mainly induced by TNF-α and IL-1 α, while A1 type astrocytes lose their normal morphology and function, can secrete neurotoxins, and have components such as C3 that mediate synaptic elimination, leading to synaptic reduction and inducing neuronal apoptosis [82]. Synapses are associated with memory processes, and a reduction in synapse number or function may contribute to memory deficits associated with AD. IL-4 and IL-10 have been demonstrated to induce the production of A2 type astrocytes, which retain their phagocytic function and capacity to secrete neuroprotective substances such as TGF-β. This has been shown to exert neuroprotective effects on neurons. Additionally, it has been demonstrated to facilitate cell proliferation, promote synapse formation, and inhibit cell apoptosis. Studies have shown that estrogen can promote the transformation of A2 phenotype and reduce the transformation of A1 phenotype, thereby protecting synapses and neurons. Both phenotypes of astrocytes show an increase in GFAP expression, and the transformation of A1 and A2 glial cells may not be independent, but a continuous process. Their proportion is related to pathological changes and the degree of cognitive impairment.

### 2.2 PD

PD is a neurodegenerative disease that is caused by the death of dopaminergic (DA) neurons in the substantia nigra pars compacta (SNpc). It is the second most prevalent neurodegenerative disease worldwide, with a prevalence rate only surpassed by AD [83,84]. However, the precise pathogenesis of PD remains unclear. The extant literature and reports have demonstrated that PD is characterized by a number of factors, including oxidative stress [85–87], calcium homeostatic imbalance [88–90], abnormal accumulation of alpha synuclein [91,92], impaired mitochondrial function [93–95], endoplasmic reticulum stress [96–98], intestinal flora dysbiosis [99,100], intestinal flora dysregulation, and many other factors. Recent studies have demonstrated that neuroinflammation plays a pivotal role in the pathogenesis of PD. In the absence of neuroinflammation, the brain is capable of removing toxins through a process known as the glymphatic system. However, excessive levels of neuroinflammation can lead to the sustained degeneration and apoptosis of dopaminergic neurons [101,102].

The brain contains a considerable number of glial cells, which have an immune effect and can produce oxidative stress and inflammation. In normal conditions, oxidative stress and inflammation have a protective effect on brain tissue. Nevertheless, aberrant activation of glial cells can result in the generation of a considerable number of free radicals and inflammatory factors, which in turn can cause severe inflammation and oxidative stress, ultimately leading to damage of brain tissue [103–111]. The high concentration of microglia in the substantia nigra region renders the substantia nigra region more susceptible to inflammation. Microglia initiate the neuroinflammatory response following the recognition of lipopolysaccharide (LPS), heat shock protein, and other stimuli, releasing inflammatory factors that initially affect DAergic neurons, resulting in neuronal stress and oxidative damage. Concurrently, the activation of astrocytes through TLR2 receptors intensifies the inflammatory response, thereby exacerbating neuroinflammation in PD [112,113]. It has recently been demonstrated that neuroinflammation, defined as the activation of microglia and astrocytes in the brain, can result in the induction of a pro-inflammatory programmed cell death pathway. This pathway is induced by caspase family proteins and is termed necroptosis. It is a necrotic and inflammatory programmed apoptotic cell death pathway [114,115]. This pathway is closely associated with neuroinflammation, a process whereby glial cells in the brain respond to inflammatory factors secreted by immune cells. When glial cells detect these factors, they regulate the inflammatory response in the CNS, secrete proinflammatory factors, increase the expression of Iba-1 and GFAP in the brain, exacerbate oxidative damage, and induce necrotic apoptosis in DAergic neurons [116–118].

The pathological features of PD are primarily characterized by the formation of Lewy bodies, which are intracellular inclusions resulting from the aberrant aggregation of α-synuclein [119]. In addition, there may be a loss of dopaminergic neurons and neurotransmitters in the SNpc and striatum of the midbrain. The substantia nigra of the midbrain is the core area of pathological changes in PD, and the degeneration and loss of dopaminergic neurons are key to the occurrence of PD [120]. The striatum is a crucial region that receives projections from dopaminergic neurons in the substantia nigra, and a decrease in dopamine directly affects the function of the striatum, leading to symptoms such as motor disorders. Moreover, the basal ganglia are the part of the brain responsible for coordinating body movements and controlling muscles. PD can cause dysfunction of the basal ganglia, which in turn affects the patient’s motor abilities.

### 2.3 HD

HD is an autosomal dominant, progressive neurodegenerative disorder with a distinctive phenotype. The primary pathological feature of HD is the production of mutant Huntington proteins, which result from the mis-expression of polynucleotide repeat sequences on the Huntingtin (Htt) gene on the patient’s chromosome 4 [121,122]. It has been demonstrated that the normal Htt protein performs multiple functions in neurons, including the maintenance of primitive neural stem cell lineage potential. In contrast, studies have demonstrated that Htt protein variants are responsible for the observed dysfunctions [123–125]. The high expression of Htt protects cells of other CNS origins from lethal injury. However, if overexpressed Htt accumulates in nerve cells to form aberrant Huntington proteins, it can lead to the development of HD, affecting the ability of nerve cells to function properly. In patients, mutant proteins typically result in damage to predominantly striatal neurons [126–132].

The pathogenesis of HD is closely related to neuroinflammation. Impairment of Htt clearance in the brain results in the accumulation of abnormalities, which in turn leads to overactivation of microglia. This leads to the clearance of the abnormal accumulation of proteins by microglia, which in turn causes neuroinflammation and dysfunction of the ubiquitin protease system and autophagy system in the brain [133,134]. Additionally, the overactivation of microglia can also result in neuroinflammation by activating NLRP3 inflammatory vesicles and secreting substantial quantities of inflammatory factors, which can cause damage to mHTT nerve cells and neurodegeneration. Furthermore, the overactivation of microglia results in the dysfunction of the ubiquitin-proteasome and autophagy systems, which in turn accelerates the progression of HD [135,136].

## 3 Neuroinflammation pathways

### 3.1 NF-κB signaling pathway

The NF-κB signaling pathway represents a prototypical inflammatory signaling pathway. The NF-κB family comprises five members: p65 (RelA), RelB, c-Rel, p50 (NFκB1), and p52 (NFκB2) [137]. The NF-κB signaling pathway is regulated by a homology domain, RHD, which binds to form a dimer and is involved in the regulation of the NF-κB signaling pathway [138,139]. The activation of the NF-κB pathway has been demonstrated to play a role in a number of biological processes, including inflammatory responses [140], cell proliferation [141], cell differentiation [142], and immune response [143]. The activation of this pathway can be classified into two categories: classical and non-classical. The classical activation pathway is associated with functions related to inflammation [144]. From a physiological perspective, NF-κB is repressed by IκB binding and is predominantly localized to the cytoplasm. Upon stimulation by bacteria, inflammation, or other stimuli, the protein kinase TAK1 is activated by pathogen-associated molecular pattern (PAMP) or damage-associated molecular pattern (DAMP) occurring in PRR-expressing immune cells. Activation of TAK1 results in phosphorylation of IKK, which subsequently releases the inhibitory effect of IκB on NF-κB, thereby promoting its activation. Activated NF-κB then transfers to the nucleus and binds to specific DNA binding sites, regulating the inflammation, apoptosis, and other responses [145,146]. The overexpression of inflammatory factors can also result in the loss of synaptic connections between neurons, impairing neuronal signal transduction and synaptic plasticity, ultimately leading to a decline in cognitive function. This process is especially evident in neurodegenerative diseases such as AD.

Some studies have demonstrated that microglia pretreated with IL-10 and subsequently stimulated with LPS exhibit a reduction in IL-6 levels. This evidence suggests that IL-10 may prevent the nuclear translocation of NF-κB, thereby reducing the transcriptional initiation of IL-6 by NF-κB. Consequently, the quantity of IL-6 is diminished, thereby attenuating the inflammatory response [147,148]. peroxisome proliferator-activated receptor alpha (PPARα) ligands have also been demonstrated to inhibit radiation-induced inflammatory responses in microglia by negatively regulating the NF-κB and AP-1 pathways [149]. These findings suggest that the interference with the nuclear translocation of NF-κB plays an important role in the attenuation of microglia inflammatory responses in neurodegenerative diseases.

### 3.2 TOLL-like receptor (TLR) signaling pathway

**Figure 1:**
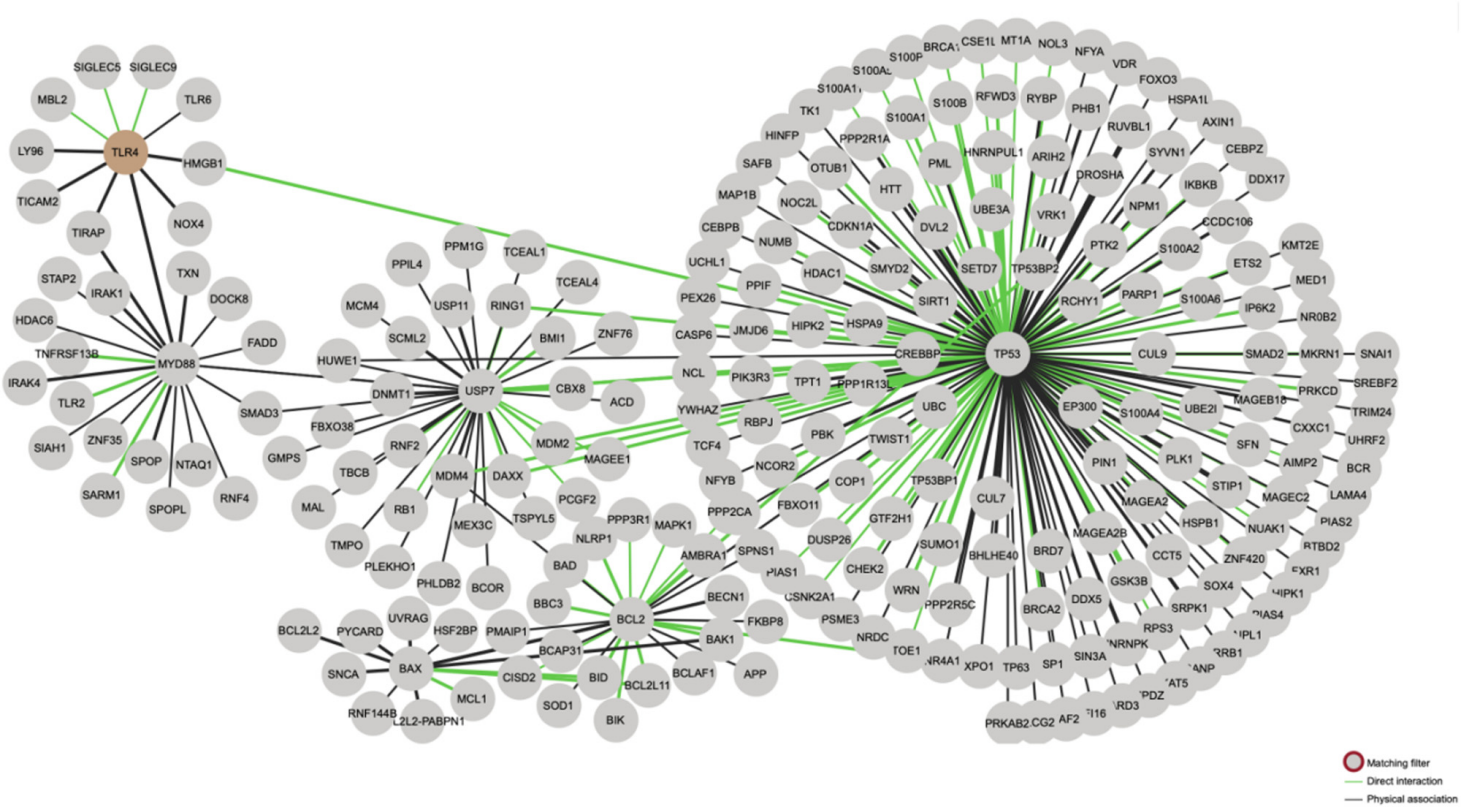
Using the String database to analyze protein interactions. TLR4 can regulate apoptosis-related proteins such as Bcl-2 and Bax through MYD88. Additionally, the P53 gene can directly regulate the TLR4 signaling pathway, as well as regulate Bcl-2 and Bax.

The MyD88 and TRIF pathways are the two pathways present in TLRs. All TLRs except TLR3 can use the MyD88 pathway, and TLR3 and TLR4 can use the TRIF pathway [150]. Activation of the TLR4 can result in the recruitment of TIR–TIRAP–MyD88 complexes, which in turn can interact with the death domain of MyD88 (Figure 1). This interaction can then lead to the recruitment of IL-1 receptor-associated kinase 4 (IRAK4) [151]. Notably, the two proteins can interact with IRAK4 also acting as an agonist to activate other proteins of the IRAK family, such as IRAK-1 [152]. This leads to the activation of TRAF6, which is activated in conjunction with E2 ubiquitin-protein ligase to activate a complex consisting of TGF-β-activated kinase 1 (TAK1), TAK1 assembly protein 1 (TAB1), TAB2, and TAB3. Ultimately the mitogen-activated protein kinase (MAPK) and NF-κB pathways are initiated by the activation of the TAK1/TAB complex [153]. The extracellular portion of TLR3 contains a horseshoe-shaped structure that facilitates the recognition of dsRNA and plays an essential role in antiviral immunity [154]. This structure then recruits the junction protein TRIF to the dsRNA. Further activation of TBK1 and RIP1 kinase forms a complex that mediates the phosphorylation process of IRF3. This translocates from the cytoplasm to the nucleus and regulates the synthesis process of type I interferon. Additionally, the activation of RIP1 also leads to ubiquitination and activation of TAK1, which in turn leads to NF-κB transcription [155]. In addition, TLR7 and TLR9 also induce type I interferon production, but rely on MyD88 activation rather than IRF3 [156]. In AD, Aβ as an endogenous messenger can activate TLR4 and other receptors, causing neuroinflammation and neuronal damage [157]. Neuroinflammation promotes the deposition of Aβ and further neuronal damage, creating a vicious cycle that exacerbates the pathological changes of AD [158]. In PD, α-synuclein aggregation and oxidative stress may also activate the TLR signaling pathway, induce microglial activation and neuroinflammation, and then exacerbate the damage and death of dopaminergic neurons in PD [159]. In MS, the TLR signaling pathway plays a key role in autoimmune inflammation. The TLR pathway can activate the infiltration and activation of immune cells such as T cells and B cells, which can lead to neuroinflammation and demyelinating lesions, thus exacerbating the pathological changes of MS [160].

### 3.3 MAPK signaling pathway

MAPK plays a prominent role as a major signaling mechanism that responds rapidly to a wide range of environmental changes and influences a variety of physiological mechanisms. The MAPK family comprises four major members: p38, extracellular signal-regulated protein kinase (ERK), c-Jun N-terminal kinase (JNK), and ERK5 [161]. The MAPK signaling pathway plays a variety of roles in various biological processes, including growth and development, oxidative stress, anti-inflammatory responses, and endoplasmic reticulum stress [162]. MAPK signaling pathways play a multitude of roles in diverse biological processes, including growth and development, oxidative stress, anti-inflammatory responses, and endoplasmic reticulum stress. The JNK–p38MAPK signaling pathway is primarily implicated in apoptosis and stress response, whereas the ERK–MAPK signaling pathway is associated with cell proliferation and differentiation. It is also inextricably linked to the cellular signaling network [163].

*In vivo*, oxidative stress stimulates the generation of ROS, which can induce the activation of ASK1, an upstream regulator of MAPK. Activated ASK1 then activates MEK4/MEK7 and MEK3/MEK6, which in turn induce the activation of JUN and P38. This activates the MAPK signaling pathway, which regulates the production of inflammatory factors and the inflammatory response *in vivo*. The activated MAPK signaling pathway is capable of regulating the production of inflammatory factors and the inflammatory response in the body. Additionally, it exerts anti-inflammatory and antioxidant effects on TLR receptors and macrophages [164]. The MAPK signaling pathway has been demonstrated to regulate the production of inflammatory factors in the body. In addition, in neurodegenerative diseases such as AD, the activated MAPK signaling pathway can also act on the NF-κB signaling pathway, promoting the release of TNF-α and IL-1β inflammatory factors to regulate the inflammatory response and further exacerbate neuronal damage in neurodegenerative diseases [165]. JNK is a critical component of the MAPK pathway, which can suppress the expression of c-Jun transcription factors and influence the genetic balance between c-Jun and AP-1. This makes it an important player in the pathway. The P38 pathway is considered to be the foundation of MAPK signaling, which is capable of responding to a multitude of environmental stimuli, and thus plays a pivotal role in the diverse functions and behaviors of the cell. ERK5 can be stimulated by a variety of external stimuli. Research has shown that ERK5 is effective in increasing insulin levels in neurons, thereby improving cell viability. It may therefore be an effective means of treating chronic degenerative brain diseases [166]. Some studies have demonstrated that ERK5 is an effective means of increasing insulin levels in neurons, thereby improving cell viability. This may have implications for the treatment of chronic degenerative brain diseases.

In neurodegenerative diseases, extracellular stimuli (such as inflammatory factors, oxidative stress products, etc.) can activate the MAPK pathway. For example, in AD, abnormal metabolism of amyloid precursor protein (APP) leads to the formation and accumulation of Aβ, which in turn triggers inflammatory responses and oxidative stress, leading to abnormal activation of the MAPK pathway [167]. The activated MAPK pathway further activates its downstream substrates, such as transcription factors and protein kinases, through phosphorylation, and then regulates the expression of inflammation-related genes [168]. In addition, the MAPK pathway can also regulate the expression of inflammation-related factors such as cyclooxygenase-2 (COX-2), inducible nitric oxide synthase (iNOS), and TNF [168]. In neurodegenerative diseases, activated MAPK signaling may participate in the pathological process of the disease by promoting neuronal apoptosis or necrosis. Abnormal activation of the MAPK pathway can lead to apoptosis and necrosis of dopaminergic neurons, thereby accelerating the progression of the PD [169]. Given the important role of the MAPK pathway in neuroinflammation and neurodegenerative diseases, intervention strategies targeting this pathway provide new ideas for disease treatment.

### 3.4 PPAR signaling pathway

PPAR is a group of nuclear receptors in the nuclear receptor family *in vivo*, including PPARα, PPARβ/α, and PPARγ. PPAR is a transcription factor that plays a pivotal role in the inflammatory response and immune regulation by regulating the metabolic and anti-inflammatory effects of transcription factors. It functions by inhibiting the release of inflammatory cytokines, adhesion molecules, and extracellular matrix proteins. Additionally, it exerts a protective effect on nerves by releasing anti-inflammatory factors that are neuroprotective. It is therefore of great importance for the recovery of cognitive function in neurodegenerative diseases. It has been demonstrated that the administration of the PPARα receptor agonist GW7647 prior to the induction of an inflammatory response in microglia results in a reduction in the phosphorylation of the AP-1C-JUN subunit, which subsequently leads to a decline in nuclear NF-kB activity [170,171]. This process slows down the inflammatory response of microglia and consequently reduces neuronal damage. PPAR-γ receptor agonists have been demonstrated to inhibit the expression of surface antigens, enhance the synthesis of NO, and decrease the secretion of prostaglandins, inflammatory factors, chemokines, and ROS. Consequently, the inhibition of PPARs expression exerts an inhibitory effect on both oxidative stress and inflammatory responses in microglia [172]. In addition, in neurodegenerative diseases such as AD, abnormal accumulation of proteins (such as Aβ) is the main cause of neuronal damage. The activation of PPARs signaling pathway may help to regulate the metabolism and clearance of these proteins to alleviate disease progression.

### 3.5 Notch signaling pathway

The Notch signaling pathway plays a pivotal role in the growth and development of astrocytes, oligodendrocytes, and dopaminergic neural precursors. Notch receptors bind to a variety of ligands and regulate the differentiation and development of cells, tissues, and organs. Activation of the Notch signaling receptor induces an inflammatory response in microglia, which is mediated by binding to the ligand Jagged1. This process ultimately leads to the production of pro-inflammatory factors by microglia, which can cause neuronal damage [173]. The activated Notch signaling pathway has been demonstrated to increase macrophage sensitivity to γ-interferon, promote inflammatory responses, and promote nuclear translocation of NF-kB, thereby exacerbating inflammatory responses. The inflammatory mediators produced can also activate the Notch signaling pathway. The activation of the Notch signaling pathway and the subsequent response involves a variety of aspects, and thus may be a potential protocol for studying early-onset neuroinflammation in neurodegenerative diseases [174]. It has been demonstrated that in neurodegenerative diseases such as AD, abnormalities in the Notch signaling pathway may contribute to pathological changes in neurons, including the formation of NFTs and plaques [175]. These changes may subsequently influence neuronal proliferation, differentiation, and apoptosis, thereby affecting neuronal survival and number. Furthermore, the Notch signaling pathway plays a role in neurodegenerative diseases, where pathological changes are exacerbated by reduced synaptic plasticity and disruption of neuronal networks [175]. This, in turn, leads to cognitive dysfunction and behavioral abnormalities.

### 3.6 PI3K/Akt signaling pathway

PI3K is a critical anti-apoptotic regulator that has been classified into three distinct types (I–III) based on its structural and regulatory characteristics. Among these, type I has been the subject of the most extensive research, and it is present in all cells, where it participates in the transduction of various signaling pathways. After the activation of PI3K, it can bind to the PH domain of downstream Akt, thereby exerting anti-apoptotic and regulatory functions on cell growth [176]. Akt is a serine/threonine protein kinase that mediates the anti-apoptotic effects of growth factor regulation and inactivates downstream apoptotic factors. Previous studies have demonstrated that neuroinflammation in the brain induces microglia to secrete inflammatory factors. Furthermore, the activation of the PI3K/Akt pathway can promote the expression of anti-inflammatory factors, such as IL-4 and IL-10, and inhibit the expression of pro-inflammatory factors, such as IL-6 and IL-1β. This is achieved by inhibiting the nuclear translocation of NF-κB, thus exerting its anti-inflammatory function [177,178].

Activation of the PI3K/Akt pathway has been demonstrated to inhibit the pro-apoptotic effect of BAD and other pro-apoptotic proteins by phosphorylating them, thereby protecting neurons from damage and death [179]. Meanwhile, the PI3K/Akt pathway can provide neurons with the necessary energy and nutrients by regulating metabolic pathways, including glycogen synthesis and fatty acid synthesis, thereby promoting the recovery of neuronal function in neurodegenerative diseases [179,180]. Reduced activity of the PI3K/Akt signaling pathway has been observed to result in pathological changes, including reduced neuronal viability, impaired synaptic plasticity, and metabolic abnormalities in AD [181]. Conversely, activation of the PI3K/Akt signaling pathway has been demonstrated to protect neurons from damage and death, thereby slowing the progression of AD [181]. Abnormalities in the PI3K/Akt signaling pathway have been linked to the death of dopaminergic neurons in PD [182]. The PI3K/Akt pathway plays a critical role in regulating neuronal apoptosis and protecting dopaminergic neurons from injury and death [182]. Therefore, it can be proposed that the PI3K/Akt signaling pathway may be a potential therapeutic target for neurodegenerative diseases.

**Figure 2:**
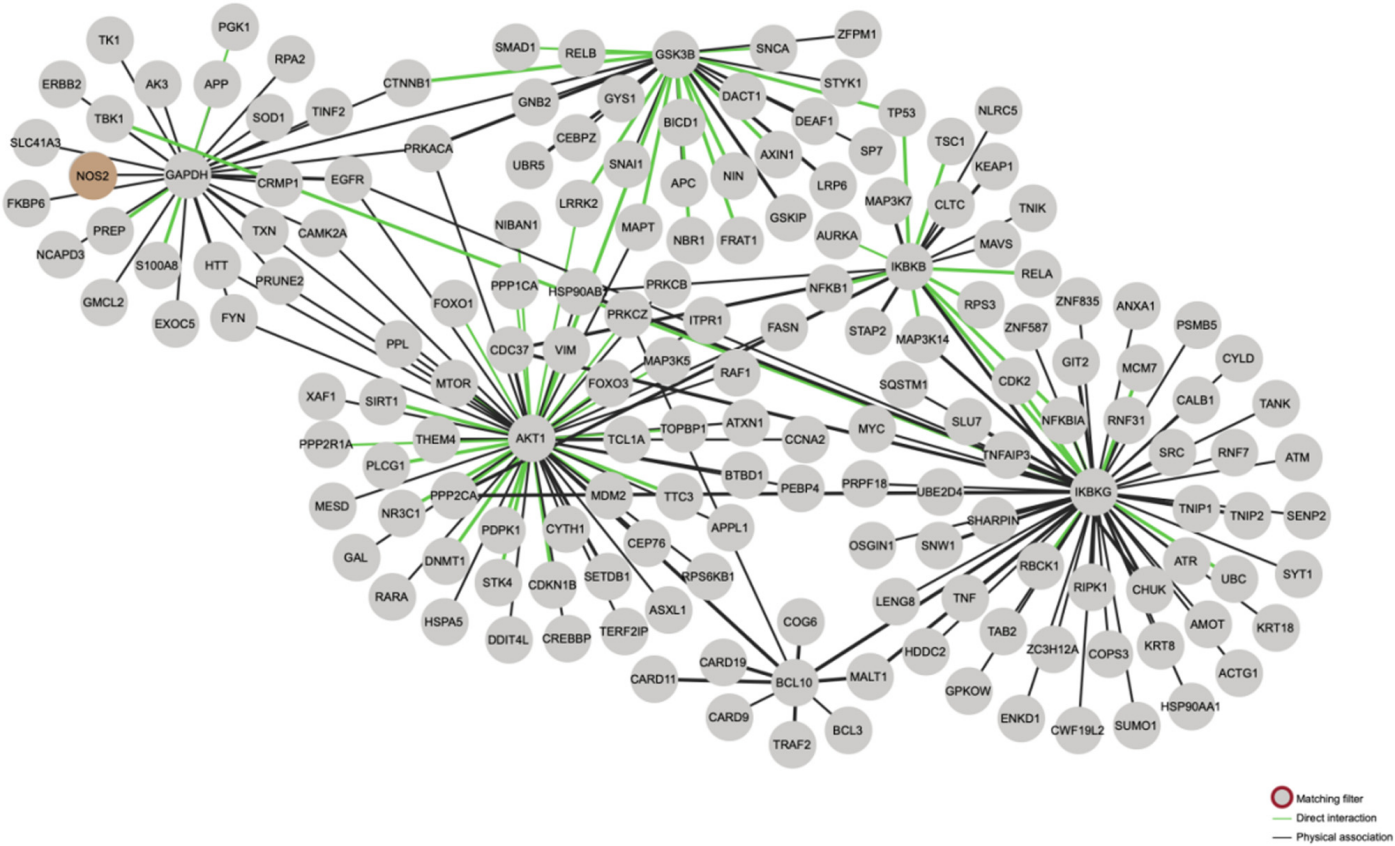
Using the String database to analyze protein interactions. The NF-κB pathway is regulated by NOS2 (iNOS), which in turn regulates TNF, Bax, and the Bcl family. It is also possible that p53 may play a role in the NF-kB pathway.

A thorough examination of the inflammation-related pathway factors revealed that numerous inflammation-related pathways interact with p53 and apoptosis-related proteins, including Bax, BID, BIK, Bak, and Bcl-2 (Figure 1). These interactions are exemplified by the well-studied iNOS or TLR family of receptors (Figure 2), which can regulate apoptosis-related proteins expression by regulating the interaction of downstream MyD88 with P53 or by directly interacting with p53 (Figure 1). In addition to the interactions between PI3K/Akt and p53, other pathways, such as NF-κB, can also influence the expression of Bax and other proteins. This is consistent with the fact that inflammation has been shown to promote neuronal apoptosis in the pathological process of neurodegenerative diseases, a phenomenon that has been the subject of increasing research in recent years.

### 3.7 Autophagy and inflammation in nervous system diseases

Autophagy represents a pivotal mechanism for cells to maintain intracellular stability and respond to diverse stress stimuli [183], with a particularly pronounced impact on nerve cells. This mechanism not only facilitates the clearance of damaged organelles and misfolded proteins, but also the removal of pathological protein aggregation [184], thereby ensuring the optimal functioning of neurons.

In neurodegenerative diseases, a defect in autophagy function is frequently a significant contributor to the accumulation of pathological proteins and the subsequent decline in neuronal function. For example, in AD and PD, the abnormal accumulation of Aβ and α-syn is closely associated with the deficiency of autophagy function [185–187]. The aggregation of these proteins not only directly damages neuronal cells, but also may trigger an inflammatory response, thereby exacerbating the damage to neurons.

In the context of neuroinflammation, activated microglia can impede the autophagy of neurons by releasing pro-inflammatory cytokines such as TNF-α and IL-1β [188]. This inhibition not only exacerbates the accumulation of pathological proteins but may also play a pivotal role in the pathogenesis of neurodegenerative diseases. Concurrently, autophagy exerts a certain anti-inflammatory effect, which can mitigate the neuroinflammatory response by eliminating inflammatory mediators in glial cells. It has been demonstrated that a deficiency in autophagy may result in the excessive activation of microglia, thereby forming a vicious circle [189]. This is to say that the inflammation that occurs in response to the initial insult exacerbates the inhibition of autophagy, which in turn further promotes inflammation.

In addition to its role in nervous system diseases, autophagy also plays an important part in the etiology of other chronic diseases. In cardiovascular diseases, autophagy is of particular importance for the survival of myocardial cells and endothelial cells [190]. This is especially the case in myocardial ischemia–reperfusion injury, where autophagy can remove damaged organelles and deal with metabolic pressure, thus protecting cardiac function. However, in contrast to its role in the nervous system, autophagy’s involvement in cardiovascular disease is more narrowly focused on cell protection and repair, with a relatively limited influence at the local cellular level [191].

In respiratory diseases, autophagy is also involved in the clearance of harmful substances, antiviral agents, and the maintenance of cellular homeostasis. Although autophagy plays an important role in lung health, the respiratory system is less dependent on autophagy than the nervous system [192,193]. In chronic lung inflammation, autophagy dysfunction may contribute to an inflammatory response and apoptosis. However, this does not directly result in neuronal degeneration, which differs from the effects observed in neurodegenerative diseases [194,195].

In conclusion, autophagy plays a complex and pivotal role in neuroinflammation and degenerative diseases. A comprehensive investigation into the regulatory mechanisms of autophagy and its specific role in various diseases is anticipated to yield novel insights and strategies for the treatment of neurodegenerative diseases and other chronic illnesses.

### 3.8 Mitosis and inflammatory in nervous system diseases

As the core mechanism of cell division, mitosis is of great importance in maintaining the homeostasis of various tissue functions. In the nervous system, this mechanism is observed to exhibit a distinctive degree of complexity. Neurons, as highly specialized cells, typically lack the capacity for division. However, glial cells, particularly astrocytes, demonstrate substantial proliferation in response to nerve injury and disease. This aberrant hyperplasia may not only exacerbate neuroinflammation but also accelerate the pathological process of neurodegenerative diseases [196].

Neuroinflammation is a pivotal mechanism in the pathogenesis of neurodegenerative diseases, whereby microglia and astrocytes are activated. Among these, astrocytes may play a pro-inflammatory role in neuritis, thereby exacerbating the inflammatory response [197]. In certain neurodegenerative diseases, the uncontrolled proliferation of glial cells (i.e., mitosis) results in an exacerbation of the inflammatory response, which directly endangers the survival of neurons [198]. The long-term proliferation of glial cells is accompanied by the release of pro-inflammatory factors, which presents a significant challenge to the function and survival of neurons. Ultimately, this may result in the death or functional decline of neurons.

It is important to highlight that the capacity for nerve regeneration in neurodegenerative diseases is also significantly influenced by the mitotic mechanism [199]. It has been demonstrated that neuritis can impede the proliferation of neural stem cells, constrain nerve regeneration, and accelerate the progression of the disease [200]. In particular, following a brain injury, this limitation of regenerative capacity is of significant consequence [201].

The proliferation of glial cells in AD and PD exhibits distinct pathological characteristics, contingent on the specific disease process. In AD, the excessive activation of microglia promotes the release of inflammatory factors, which in turn exacerbates the accumulation of Aβ, thereby forming a vicious cycle [202]. In PD, the proliferation of astrocytes may exacerbate the inflammatory response and impact the survival of neurons [25].

In contrast, cell proliferation in cardiovascular diseases (such as smooth muscle cells) and airway smooth muscle and fibroblast proliferation in respiratory diseases (such as COPD) are also involved in the process of cell proliferation. However, their primary role is tissue repair and remodeling, which does not directly result in the loss of neural function [203]. For example, in atherosclerosis, the proliferation of smooth muscle cells is essential for vascular repair; however, excessive proliferation may result in the thickening of the vascular wall, further obstructing blood flow, and ultimately leading to vascular sclerosis [204]. In COPD, the proliferation of airway smooth muscle and fibroblasts may result in airway remodeling and an increase in lung injury. However, these proliferation reactions are more closely associated with tissue repair [205,206].

The neuroinflammation and functional loss caused by glial cell proliferation in neurodegenerative diseases are more persistent and destructive, and are difficult to reverse [8]. This not only elucidates the pivotal role of glial cells in neuroinflammation and neurodegenerative diseases but also identifies a crucial target for future treatment strategies. A comprehensive investigation of the regulatory mechanisms governing glial cell proliferation may facilitate the development of novel therapeutic strategies for neurodegenerative disorders (Table 2).

**Table 2:** Summary of pharmacology related to inflammatory signaling pathways in neurodegenerative diseases

Signaling pathways	Related neurodegenerative diseases	Primary mechanism of action	Pharmacological intervention strategies	Known drugs or treatments	References
Notch signaling pathway	AD, HD	Involved in neuronal differentiation and neural stem cell renewal, regulating neural development and synaptic function	Inhibits Notch signaling pathway to regulate the course of neurodegenerative diseases	Gamma-secretase inhibitors (e.g., DAPT)	[207–209]
mTOR signaling pathway	AD, PD, HD	Involved in neuronal metabolism and clearance by controlling processes such as protein synthesis, cell growth, and survival	Promotes autophagy and removal of damaged proteins by inhibiting the mTOR signaling pathway	Rapamycin	[210–212]
JAK/STAT signaling pathway	AD, PD	Involved in immune and neuroinflammatory responses that may exacerbate neurodegenerative pathologies	Inhibit JAK/STAT pathway to reduce neuroinflammation	JAK inhibitors (e.g., Tofacitinib)	[213,214]
NF-κB signaling pathway	AD, PD, HD	Modulation of inflammatory response, involved in neuroinflammation and apoptosis	Inhibit NF-κB signaling pathway to reduce neuroinflammatory response	Curcumin	[215–217]
PI3K/Akt/mTOR pathway	AD, PD	Influences neuronal survival and stress response by regulating processes such as cell survival, proliferation, and metabolism	Activate PI3K/Akt pathway to enhance neuroprotection and anti-stress ability	Lysophosphatidic acid	[218,219]

## 4 Immune cells, receptors, and factors related to neuroinflammation

Neuroinflammation is an important pathological process in neurodegenerative diseases, with various factors, including immune cells, cytokines, chemokines, and receptor factors, playing a pivotal role in its mediation. These inflammatory mediators interact with each other to jointly promote neuronal cell damage, functional disorders, and even apoptosis, thereby driving the occurrence and development of neurodegenerative diseases.

### 4.1 Immune cells

Microglia are resident immune cells in the CNS, constituting approximately 10% of CNS cells and 20% of glial cells in the brain [232,233]. In their resting state, microglia assist in the detection of subtle alterations in pathogens and the microenvironments, acting as sentinels to recognize a multitude of molecular patterns through surface receptors. Upon the perception of damage or PAMPs and DAMPs, microglia undergo a rapid activation process, transitioning from a quiescent state to an activated state and subsequently releasing a variety of inflammatory mediators [234,235]. Astrocytes also play a significant role in neuroinflammation, as they are capable of releasing inflammatory mediators and participating in the regulation of neuroinflammation by altering cell morphology and function [236]. Furthermore, activated astrocytes can form glial scars, which to some extent limit the spread of inflammation [236]. However, excessive glial scar formation may also hinder nerve regeneration. Macrophages have the capacity to migrate from the peripheral blood to the nervous system, participate in inflammatory responses, release inflammatory mediators, and engulf pathogens or damaged cells [237]. In certain neuroinflammatory diseases, such as MS, T cells and B cells contribute to the occurrence and development of neuroinflammation by recognizing self-antigens or foreign antigens, thereby activating immune responses [238].

### 4.2 Cytokines

As crucial signaling molecules in the neuroinflammatory system, cytokines include a variety of types, the most representative of which include TNF-α, IL-1β, and IL-6 [239]. These cytokines play a pivotal role in the complex immune network, mainly released by activated immune cells such as microglia, macrophages, and certain T cells and B cells [240,241]. In the pathophysiology of neuroinflammation, they are not only the key mediators of early responses, but also profoundly affect the subsequent developmental trajectory of inflammation. Specifically, TNF-α, as a potent pro-inflammatory cytokine, can trigger a series of cascade reactions, promote the activation of vascular endothelial cells, increase vascular permeability, and allow more immune cells and inflammatory mediators to enter the site of inflammation [242]. At the same time, TNF-α can also activate the expression of other cytokines and chemokines, further aggravating the neuroinflammatory response [243]. IL-1β mainly activates transcription factors such as NF-κB, upregulates the expression of adhesion molecules and pro-inflammatory mediators, and promotes the adhesion and migration of inflammatory cells [244]. It plays a particularly significant role in neuroinflammation, inducing the activation of neurons and glial cells, triggering excitotoxicity in neurons, and even leading to neuronal death [244]. IL-6 exhibits more complex functions. On the one hand, it can serve as an acute-phase protein and participate in the body’s defense response; on the other hand, in persistent or excessive inflammatory responses, the overproduction of IL-6 may be closely related to the pathogenesis of various autoimmune diseases and neurodegenerative diseases [245]. It can finely regulate the immune response by regulating the proliferation and differentiation of T cells and B cells, as well as promoting the synthesis of acute-phase proteins by liver cells [245]. These cytokines play a key role in the initiation and maintenance of neuroinflammation by promoting the activation and recruitment of inflammatory cells and enhancing the immune response.

### 4.3 Chemokines

As a class of cytokines that can specifically guide immune cells (such as leukocytes, monocytes, macrophages, etc.) to migrate to the site of inflammation, chemokines play an indispensable role in the process of neuroinflammation. They bind to CCRs on the surface of cells, triggering intracellular signaling pathways, thereby guiding the directional movement of cells. Monocyte chemoattractant protein-1 (MCP-1), as an important member of the chemokine family, is particularly noteworthy [246]. During the onset of neuroinflammation, the expression of MCP-1 is significantly upregulated, attracting monocytes to cross the blood–brain barrier and enter the CNS, further promoting the exacerbation of inflammatory reactions [221]. The receptor factors associated with neuroinflammation mainly include PRRs and CCRs [246]. Among them, PRRs mainly include TLRs, purinergic receptors, etc., which can recognize PAMPs or DAMPs to trigger immune responses [247]. CCRs such as CCR2 can bind to chemokines and direct immune cells to the site of inflammation [248].

### 4.4 Oxidative stress and other factors

ROS, as a product of oxidative stress, also play a critical regulatory role in neuroinflammation. The excessive production of ROS can disrupt the redox balance within cells, leading to oxidative damage to neurons and glial cells, which in turn can cause cellular dysfunction and even death [249]. This oxidative stress state not only directly exacerbates the severity of neuroinflammation, but also may indirectly promote the expression of chemokines and the migration of immune cells by activating related signaling pathways. On the other hand, NO and prostaglandins, as important signaling molecules in the body, also participate in the regulation of neuroinflammation [250]. NO, through its free radical properties, can affect various physiological and pathological processes such as vascular permeability, cell proliferation, and apoptosis, thereby influencing the occurrence and development of neuroinflammation [250]. Prostaglandins are a class of lipid mediators with a wide range of biological activities [250]. They have a profound effect on the neuroinflammatory process by regulating vascular relaxation, pain perception, and recruitment of inflammatory cells in inflammatory responses.

## 5 Neuroinflammation-induced apoptosis

**Figure 3:**
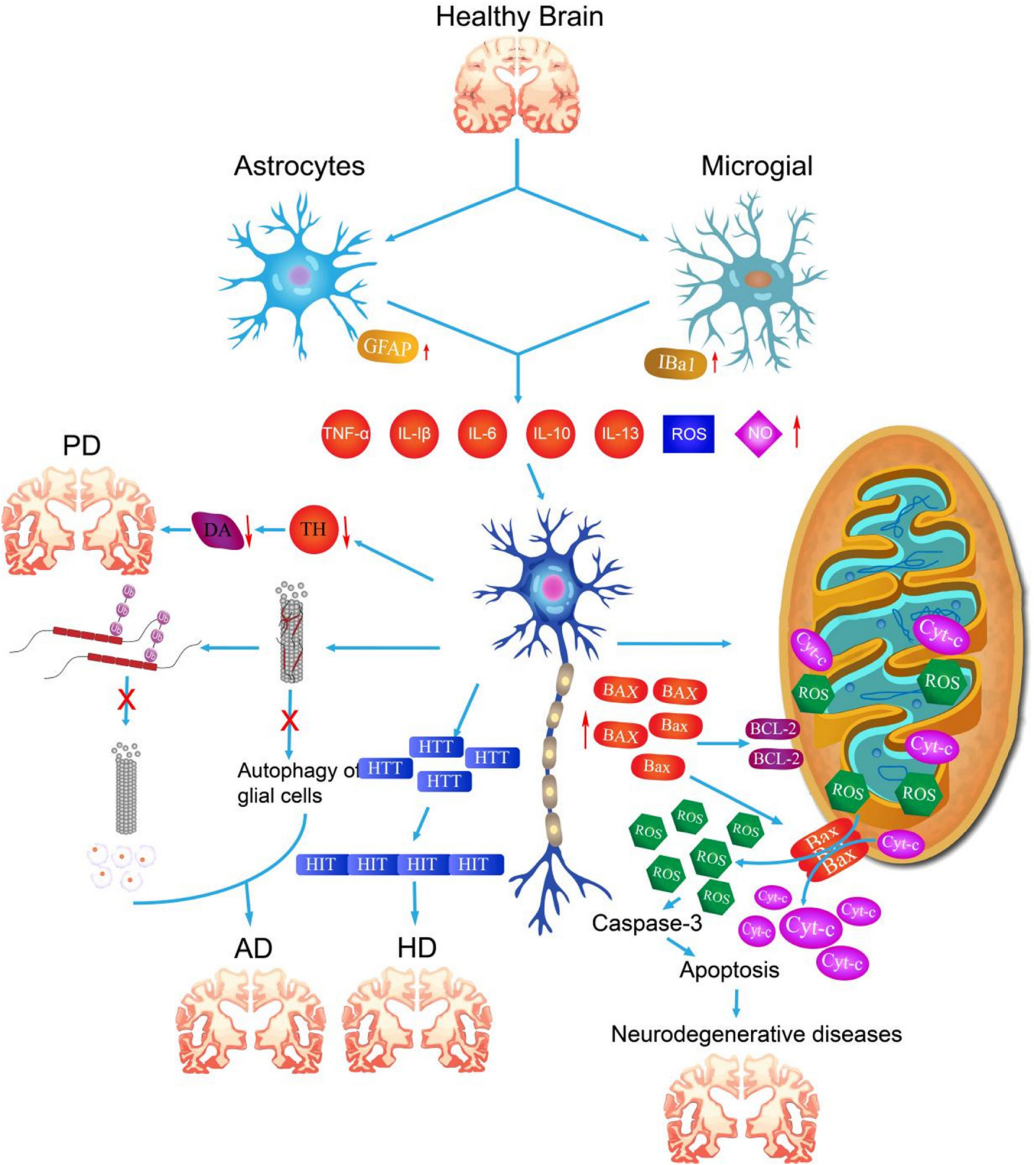
Occurrence and development of neurodegenerative diseases induced by neuroinflammation. Due to the influence of environmental stimuli, aging or genetic factors, neuroinflammation in the brain is induced, and further immune cells release excessive inflammatory factors, which stimulate the changes in the levels of various degenerative markers of neuron cells, abnormal mitochondrial metabolism, and htt gene mutation, leading to the occurrence of PD, AD, HD, and other neurodegenerative diseases.

Neuroinflammation in neurodegenerative diseases leads to apoptosis of neurons by producing inflammatory mediators, exacerbating oxidative stress, increasing the concentration of glutamate in the intercellular space, regulating the cell death mode, the direct effect of immune cells, and destroying the blood–brain barrier. Apoptosis is a genetically controlled process of programmed cell death. It is a process of cell death carried out by the organism to maintain the stability of the internal environment. Morphologically, the principal manifestations are the crumpling of the nucleus, as well as the degradation of the chromosomal DNA of the cell, the rupture of the nucleolus, the formation of vesicles in the cytosolic membrane, and the gradual division of the cell into several apoptotic vesicles, which are ultimately absorbed by phagocytes [251,252]. These different pathways of neuroinflammation interact to form a complex network that leads to the neuronal apoptosis and then promotes the onset and development of neurodegenerative diseases (Figure 3 and Table 3).

**Table 3:** Relationship between neuroinflammation regulated by apoptosis pathway and neurodegenerative diseases

Apoptosis pathway	Neurodegenerative diseases	Degree of association	Associations	References
Mitochondrial pathway	AD	High	Impaired mitochondrial function in the brain of AD patients leads to the activation of apoptosis-related proteins (e.g., Caspase-9), which accelerates neuronal death.	[220]
	PD	High	In PD, mitochondrial dysfunction promotes dopaminergic neuronal death through oxidative stress and caspase-dependent pathways.	[221]
	HD	Medium	In HD, mitochondrial damage exacerbates intracellular Ca²⁺ accumulation, which in turn activates apoptotic pathways.	[222]
Fas/FasL pathway	AD	Medium	Hyperactivation of the Fas/FasL signaling pathway in the brains of AD patients promotes neuronal death.	[223]
	ALS	Medium	The Fas/FasL pathway in ALS plays an important role in the apoptotic process of motor neurons.	[224]
	HD	Medium	In HD, over-activation of Fas receptors is closely associated with nerve damage.	[225]
Notch pathway	AD	Medium	Aberrant activation of the Notch signaling pathway is associated with neuronal injury and amyloid deposition. Notch interacts with APP proteins and may exacerbate the aberrant processing of APPs, which promotes the formation of amyloid plaques and affects neuronal cell survival.	[226]
	PD	Medium	The Notch pathway may have an impact on neurodegenerative processes by regulating the production of neuroprotective factors and their ROS. Studies have shown that proper regulation of Notch signaling helps protect dopaminergic neurons from oxidative stress-induced injury.	[227]
	HD	low	Evidence suggests that Notch may have a slight effect on disease course by regulating intracellular Htt protein development and neuronal survival signaling.	[228]
p53 pathway	AD	High	p53 plays an important role in AD by promoting neuronal apoptosis, and its expression correlates with AD progression.	[229]
	PD	High	p53 promotes disease progression by inducing apoptosis and oxidative stress in neuronal cells in PD models.	[230]
	HD	Medium	The p53 pathway acts in HD by regulating neuronal death and mitochondrial functional response.	[231]

### 5.1 Mitochondrial pathway apoptosis

The mitochondrial pathway is one of the important pathways of neuronal apoptosis induced by neuroinflammation. The regulation of mitochondrial apoptosis is primarily attributed to the Bcl-2 family, which is divided into two major classes based on its function. These classes include anti-apoptotic and pro-apoptotic proteins, which work in concert to maintain the stability of mitochondria [253]. The pro-apoptotic proteins contain the BH3 domain and are categorized as Bak, Bax, Bok, Bim, Bad, Bid, Bik, and Bmf. Antiapoptotic proteins include Bcl-2, Bcl-xl, Bcl-W, and others, which contain the BH4 domain [254]. These anti-apoptotic proteins mainly distributed in the mitochondrial membrane, and can stabilize the mitochondrial membrane potential, inhibit the activity of pro-apoptotic proteins, and maintain the normal functions of mitochondria [255,256].

**Figure 4:**
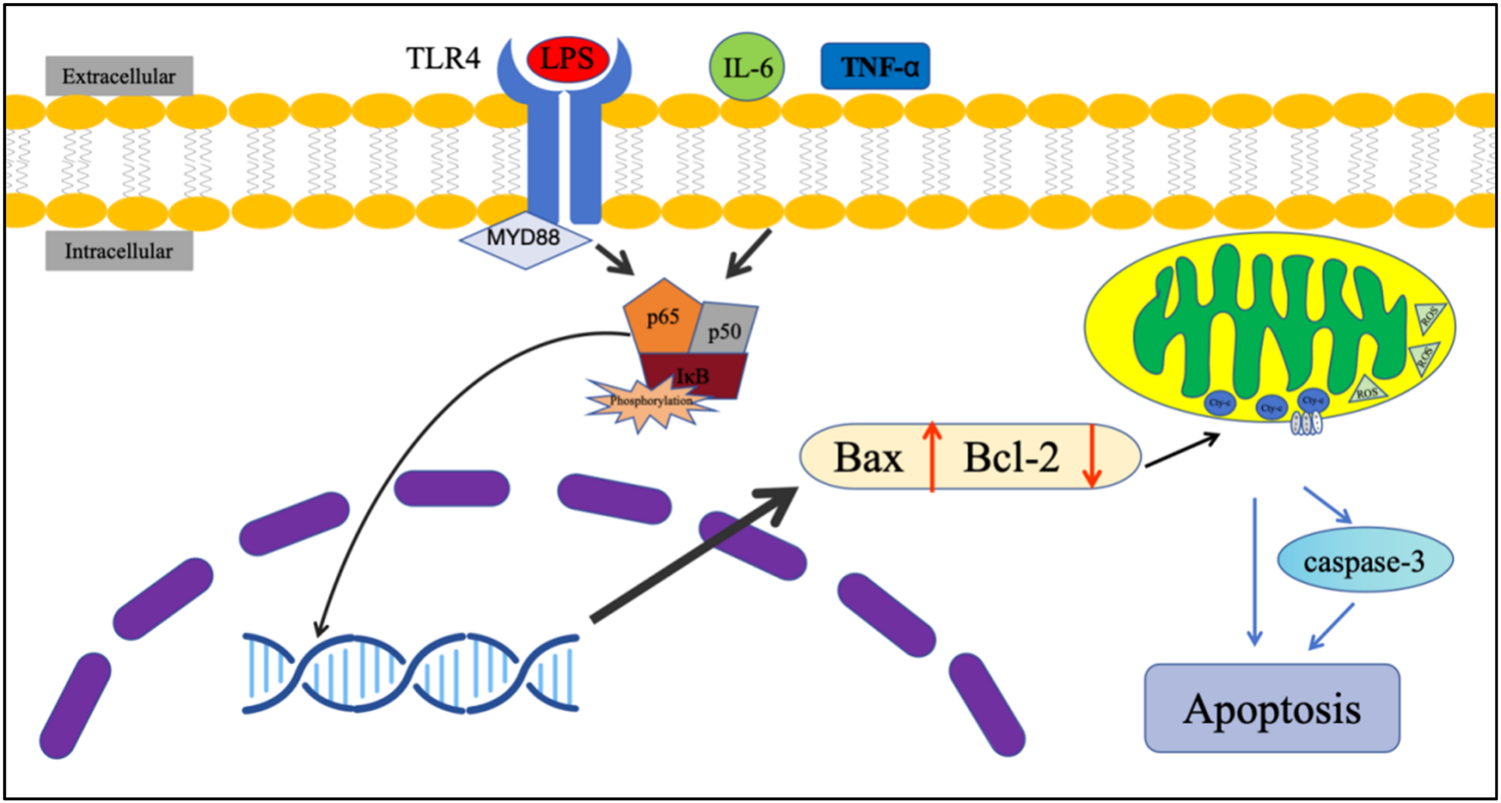
Schematic diagram of the signaling pathway of LPS-induced apoptosis. LPS, as well as inflammatory factors, activate MYD88 by acting on the membrane receptor TLR4, thus mediating the activation of the NF-κB signaling pathway. Activated NF-κB moves to the nucleus and exerts the role of a transcription factor to regulate the expression of genes. It is also involved in regulating the expression of genes such as Bax, Bcl-2, etc., and up-regulating the expression of apoptosis-associated proteins such as Bax and down-regulating the expression level of anti-apoptotic proteins such as Bcl-2. Bax and Bak form a pore-like structure at the mitochondrial surface, which leads to the loss of structural integrity of the mitochondrial membrane and the release of contents. This results in the activation of the intracellular caspase family and the apoptosis of the cellular mitochondrial pathway.

**Figure 5:**
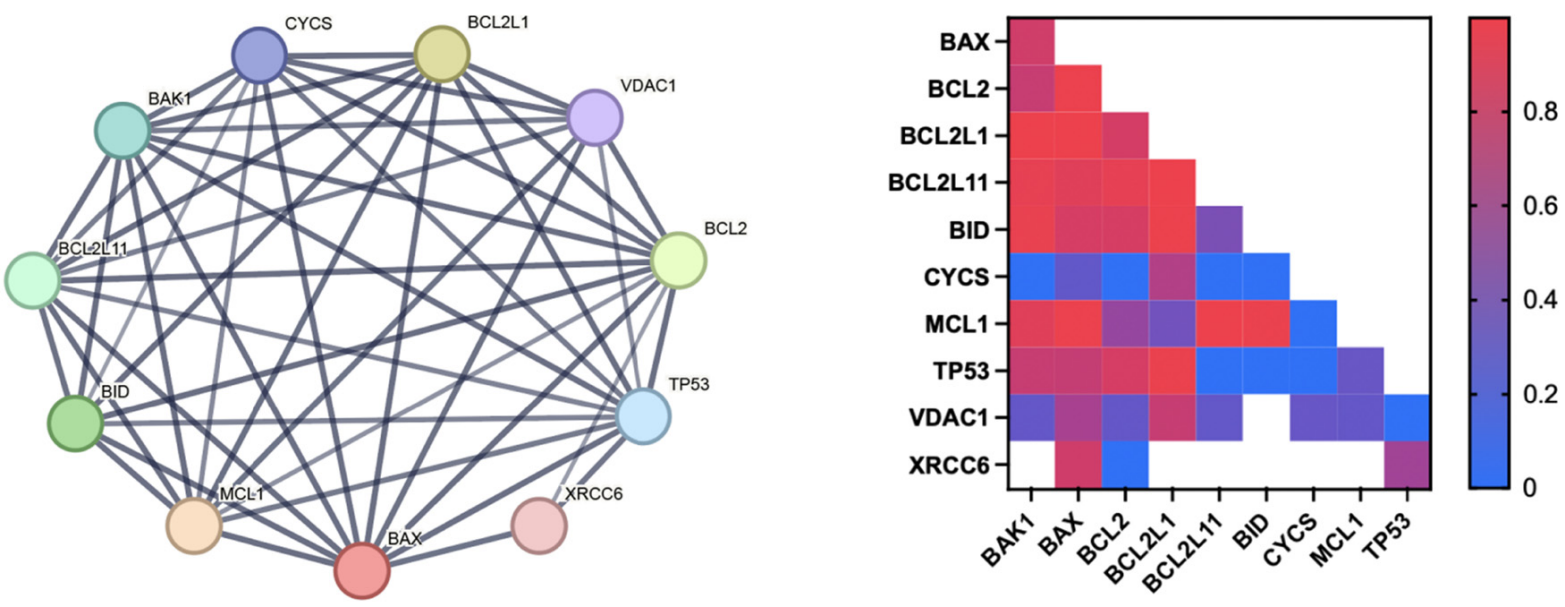
Using the String database to analyze protein interactions. A search of the String website revealed that P53 has robust interactions with apoptosis-related proteins, including Bax, Bcl-2, and Bak.

In neuroinflammatory cells associated with neurodegenerative diseases, upon receiving apoptotic signals, apoptotic proteins, such as Bak and Bax, undergo a transition from inhibition to activation, translocation, and localization to the mitochondrial membrane. They bind to apoptotic proteins such as Bcl-2 and inhibit its function. This results in the formation of transmembrane pores on the surface of the mitochondrial membrane, a reduction in the mitochondrial membrane potential, and the destruction of mitochondrial stability [257,258]. A reduction in the mitochondrial membrane potential and an imbalance in stability results in the release of mitochondrial contents, such as Cytc, ROS, and other mitochondrial contents, into the cytoplasm, activating intracytoplasmic caspase-3 to initiate the apoptotic process of neurons in an inflammatory state in neurodegenerative diseases such as PD (Figure 4) [259,260]. P53, which is highly related to mitochondrial metabolism, can be transferred to the surface of mitochondria and bind to Bcl-2, inhibiting its anti-apoptotic activity (Figure 5). It can also interact with Bax protein, resulting in increased expression of both, and promoting the apoptotic response [261,262].

### 5.2 Pyroptosis pathway

**Figure 6:**
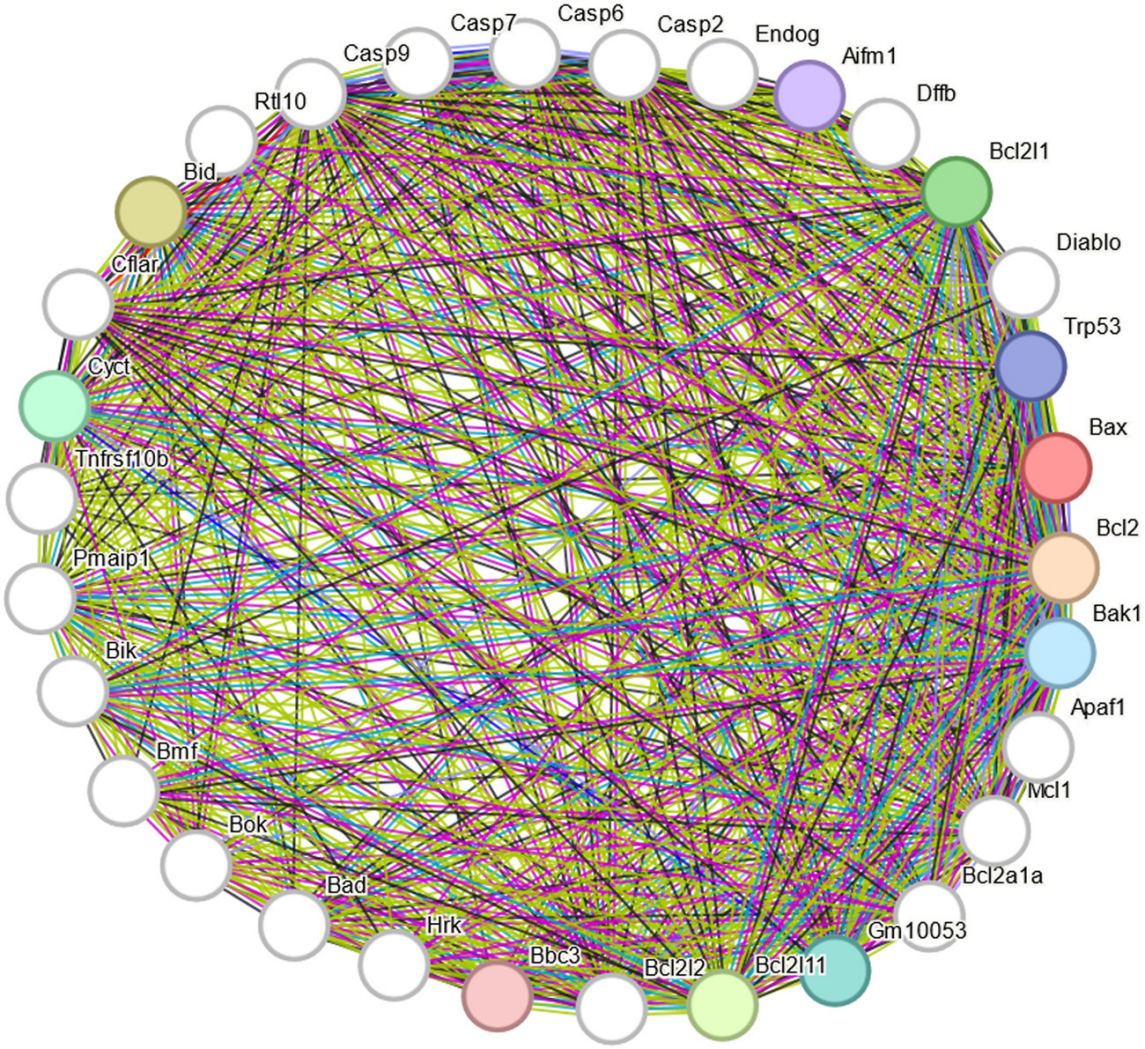
Using the String database to analyze protein interactions. P53 interacts with a number of proteins involved in apoptosis, including Bax and Bcl-2. In addition, caspase-6, caspase-7, and caspase-9, among others in the caspase family, interact with proteins involved in apoptosis, including Bax and Bcl-2.

In this article, apoptosis-related proteins such as Bax, Bcl-2, and P53 were found to be closely related to the caspase protein family associated with cellular pyroptosis by searching the STRING protein interactions website (Figure 6). Pyroptosis is a caspase-1-dependent programmed cell death pathway that results in the rupture of cell membranes and the subsequent release of large amounts of inflammatory factors. In 2005, Prof. Shao Feng’s team was the first to identify the cellular pyroptosis pathway. The main mechanism is that caspase-1/4/5/11 induces cellular pyroptosis by cleaving GSDMs [263]. GSDMs are a family of proteins with perforation effects, consisting of six types of genes. Of these, GSDMD and GSDME are the key molecules in the development of cellular pyroptosis [264–267]. The current study identified two distinct pathways for the activation of the cellular pyroptosis process: the classical and non-classical pathways.

The classical activation pathway is initiated by a class of protein complexes, designated as inflammatory vesicles, which are composed of PRRs, ASCs, and caspase-1. Activation of caspase-1 results in the maturation of pro-IL-1β and pro-IL-18, as well as cleavage of GSDMD into the small molecule proteins NT and CT. NT can bind to lipid molecules on the surface of the cell membrane, forming pores in the membrane, which results in the release of IL-1β. TNF-α and HMGB1 are released from the cell through the pores, and at the same time, water molecules enter the cell through the pore, leading to cell rupture. This further increases the release of inflammatory factors and exacerbates apoptosis and inflammatory responses [268,269].

The nonclassical pathway, also known as noncaspase-1-dependent nonclassical activation, involves direct stimulation of caspase-4/5/11 by LPS, which promotes the cleavage of GSDMD and the maturation and release of inflammatory factors [270,271]. Alternatively, the non-inflammatory apoptosis of cells can be converted to cellular pyroptosis by the activation of caspase-3, which cleaves GSDME. Furthermore, the activation of caspase-8 cleaves GSDMD, thereby switching cells from the apoptotic pathway to pyroptosis [272,273].

In the process of neuroinflammation, the pro-inflammatory factors released by activated immune cells can trigger the process of cell death. Pyrosis further exacerbates the inflammatory response, creating a vicious cycle that continuously damages neurons in neurodegenerative diseases [274]. On the other hand, cell death leads directly to the death of neurons through the rupture of the cell membrane and the release of cell contents [274]. This is an important mechanism of neuronal loss in neurodegenerative diseases. The onset and development of neurodegenerative diseases are often accompanied by neuroinflammation and cell death. For example, in AD, the abnormal accumulation of β-amyloid protein can cause an inflammatory response and then trigger cell death, leading to neuronal death. Similar inflammatory responses and cell death may occur in PD, ALS, and other neurodegenerative diseases.

Recent studies on neuronal apoptosis have provided additional insights beyond the two pathways mentioned above. For example, it has been demonstrated that this process can influence the occurrence of neuronal apoptosis by triggering the activation of NLRP3 inflammatory vesicles. Moreover, several studies have demonstrated that GSDMD and GSDME are also involved in the process of necrotic apoptosis [275,276]. In addition to apoptosis, the ubiquitin metabolism system within neurons, the autophagy system, epitope modification of DNA, and abnormal levels of intracellular lactate have been proposed as potential contributors to the development of neurodegenerative diseases [277,278]. The current comprehensive investigation of neuronal damage and apoptotic pathways offers a substantial theoretical foundation for the pursuit of neuronal apoptosis-associated neurodegenerative disease mechanisms. This will facilitate the identification of the most pivotal pathways or biomarkers influencing neurodegenerative disease progression and the development of therapeutic strategies for neurodegenerative diseases.

## 6 Perspective

As life expectancy continues to increase, aging-related neurodegenerative diseases such as PD and AD will become one of the most significant threats to the quality of healthy life in old age. Therefore, there is an urgent need to investigate the pathogenesis of such diseases and their potential possible pathogenic factors, and to explore drugs and medical treatments that can effectively treat or delay the development of the disease, so as to help patients improve their quality of life. This study reviews the characteristics and specific pathways of neuroinflammation in the early stages of neurodegenerative diseases. Nevertheless, the relationship between long-term inflammatory infiltration and neuronal cell activity, as well as the association between neuronal damage and loss and long-term neuroinflammation, remains poorly understood. These areas warrant further investigation by neuroscientists. It remains unclear whether the cellular pyroptosis pathway, which has been identified in recent years, is also involved in the pathogenesis of neurodegenerative diseases. Consequently, the relationship between long-term inflammation and neuronal damage and loss, as well as the potential role of cellular pyroptosis in neuronal loss, will likely be a significant area of investigation in the field of neurodegenerative diseases and neuroinflammation.
